# Comparing the Usefulness of Distance, Monophyly and Character-Based DNA Barcoding Methods in Species Identification: A Case Study of Neogastropoda

**DOI:** 10.1371/journal.pone.0026619

**Published:** 2011-10-24

**Authors:** Shanmei Zou, Qi Li, Lingfeng Kong, Hong Yu, Xiaodong Zheng

**Affiliations:** Key Laboratory of Mariculture Ministry of Education Ocean University of China, Qingdao, China; American Museum of Natural History, United States of America

## Abstract

**Background:**

DNA barcoding has recently been proposed as a promising tool for the rapid species identification in a wide range of animal taxa. Two broad methods (distance and monophyly-based methods) have been used. One method is based on degree of DNA sequence variation within and between species while another method requires the recovery of species as discrete clades (monophyly) on a phylogenetic tree. Nevertheless, some issues complicate the use of both methods. A recently applied new technique, the character-based DNA barcode method, however, characterizes species through a unique combination of diagnostic characters.

**Methodology/Principal Findings:**

Here we analyzed 108 COI and 102 16S rDNA sequences of 40 species of Neogastropoda from a wide phylogenetic range to assess the performance of distance, monophyly and character-based methods of DNA barcoding. The distance-based method for both COI and 16S rDNA genes performed poorly in terms of species identification. Obvious overlap between intraspecific and interspecific divergences for both genes was found. The “10× rule” threshold resulted in lumping about half of distinct species for both genes. The neighbour-joining phylogenetic tree of COI could distinguish all species studied. However, the 16S rDNA tree could not distinguish some closely related species. In contrast, the character-based barcode method for both genes successfully identified 100% of the neogastropod species included, and performed well in discriminating neogastropod genera.

**Conclusions/Significance:**

This present study demonstrates the effectiveness of the character-based barcoding method for species identification in different taxonomic levels, especially for discriminating the closely related species. While distance and monophyly-based methods commonly use COI as the ideal gene for barcoding, the character-based approach can perform well for species identification using relatively conserved gene markers (e.g., 16S rDNA in this study). Nevertheless, distance and monophyly-based methods, especially the monophyly-based method, can still be used to flag species.

## Introduction

DNA barcoding has been proposed as a method that will make species identification faster and more accessible using a small fragment of DNA sequence, particularly in species with complex accessible morphology [1], [2], [3], [4], [5], [6]. When the reference sequence library is in place, new specimens and products can be identified by comparing their DNA barcode sequences against this barcode reference library. In this sense species identification using DNA barcoding should be kept clear and distinct from other proposed uses of DNA sequence information in taxonomy and biodiversity studies, such as “DNA taxonomy” using DNA sequences [7], [8], [9]. So far, DNA barcoding has gained wide popularity with well over 1,000 publications involving it [10].

Presently, most methods of DNA barcoding are tree-based and can fall into two broadly defined classes. One (distance-based) is based on degree of DNA sequence variation within and between species. Another (monophyly-based) requires the recovery of species as discrete clades (monophyly) on a phylogenetic tree [2]. The distance-based approach converts DNA sequences into genetic distances and then uses these distances to establish identification schemes. This approach defines a similarity threshold below which a DNA barcode is assigned to a known or a new species. Several authors (e.g., [11], [12]) also proposed the notion of a “barcoding gap”, a distance-gap between intra- and interspecific sequences [13], [14], [15], for species identification. However, the distance-based approach seems to be ill suited as a general means for species identification and the discovery of new species [9], [16], [17]. One reason is that substitution rates of mitochondrion DNA vary between and within species and between different groups of species. The varied substitution rates can result in broad overlaps of intra- and interspecific distances [9], [17], [18], [19] and hinder the accurate assignment of query sequences [13], [20], [21]. The monophyly-based approach uses monophyly on a phylogenetic tree to assign unknown taxa to a known or new species. Similarly, some issues complicate the use of monophyly in a barcoding framework. For example, the long-recognized problem of incomplete lineage sorting will yield gene genealogies that may differ in topology from locus to locus [22], [23]. The recently divergent taxa may not be reciprocally monophyletic due to lack of time needed to coalesce [24], [25]. In addition, the gene trees are not necessarily congruent with species trees (e.g., [15], [26], [27]), and the monophyly, while a discrete criterion, is arbitrary with respect to taxonomic level [23], [28], [29].

A recently applied new technique, the character-based DNA barcode method, has been proposed as an alternative to tree-based approaches for DNA barcoding [16], [21], [23], [30]. This method is based on the fundamental concept that members of a given taxonomic group share attributes that are absent from comparable groups [31]. It characterizes species through a unique combination of diagnostic characters rather than genetic distances. The four standard nucleotides (A,T,C,G) if found in fixed states in one species can be used as diagnostics for identifying that species. This way, species boundaries can be defined by a diagnostic set of characters which can be increased to any level of resolution by applying multiple genes [21]. Presently, character-based DNA barcode method has been proved useful for species identification and discovery of several taxa, for example, *Drosophila* and odonates [21], [23], [32].

In this study, the usefulness of distance, monophyly and character-based barcoding approaches was tested by barcoding Neogastropoda across a broad spectrum of neogastropod species, genera, and families. The broad spectrum allowed us to estimate average divergence values and find diagnostic characters across a range of taxonomic levels. While most barcoding studies have primarily focused on a single marker gene - the mitochondrial cytochrome *c* oxidase subunit I (COI) - as a source for identifying diagnostic barcodes (e.g., [2], [11], [33], [34], [35], [36]), opponents argue the limitation of a single mitochondrial DNA gene for barcoding (e.g., [19], [37], [38]). Here two mitochondrial genes COI and 16S ribosomal DNA (16S rDNA) were used for barcoding Neogastropoda.

The order Neogastropoda (Gastropoda: Caenogastropoda) represents a species rich (approx. 16,000 living species) marine gastropod group and has adapted to almost every marine environment [39], [40]. It contains many well-known, diverse, and ecologically significant families (such as Muricidae, Buccinidae, and Conidae) and has a well-established morphological taxonomic system [40], [41], [42], [43], [44], [45]. However, the identification of neogastropod taxa is often difficult since the morphological characters (shell characters and the anatomy of the digestive system) that species identification bases on are not only varied within groups but also easily to be impacted by environment. In addition, there are lots of closely related species within different neogastropod families. Therefore, Neogastropoda provides an ideal case for contrasting the various types of DNA barcoding (distance, monophyly and character-based methods). By exploring the potential of various DNA barcoding approaches in Neogastropoda, we can get a clearer idea of how the DNA sequence information can be used in species identification.

## Materials and Methods

### Taxon Sampling

We analysed both COI and 16S rDNA sequences from 113 individuals of 40 species belonging to 25 genera and 12 families within Neogastropoda (Table S1). All neogastropod samples were collected from 31 localities along the whole China coast from 2003 to 2010 and stored in 90–100% ethanol (Table S1, Figure S1).

### DNA Extraction, PCR Amplification, and Sequencing

DNA was extracted from small pieces of foot tissue by the CTAB method as modified by Winnepenninckx et al. [46]. PCR reactions were carried out in a total volume of 50 µL, using 1.5 mM MgCl_2_, 0.2 mM of each dNTPs, 1 µM of both forward and reverse PCR primers, 10× buffer and 2.5 U *Taq* DNA polymerase. Thermal cyclings were performed with an initial denaturation for 3 min at 95°C, 45 s at primer-specific annealing temperatures (45–50°C for COI and 16S rDNA), and 1 min at 72°C, followed by 35 cycles of 30 s at 95°C, 45 s at primer-specific annealing temperatures (45–50°C for COI and 16S rDNA), 1 min at 72°C, with a final extension of 10 min at 72°C. PCR and sequencing primers for COI were LCO1490(F)–GGTCAACAAATCATAAAGATATTGG and HCO2198(R)–TTAACTTCAGGGTGACCAAAAAATCA [47]. PCR and sequencing primers for 16S rDNA were 16Sar–CGCCTGTTTATCAAAAACAT, 16Sbr–CCGGTCTGAACTCAGATCACGT [48], 16SarM–GCGGTACTCTGACCGTGCAA and 16SbrM–TCACGTAGAATTTTAATGGTCG [49]. The PCR products were confirmed by 1.5% agarose gel electrophoresis and stained with ethidium bromide. The fragment of interest was either purified directly (using EZ Spin Column PCR Product Purification Kit, Sangon), or cut out of the gel and purified when additional bands were visible (EZ Spin Column DNA Gel Extraction Kit, Sangon). Purified products were sequenced in both directions using the BigDye Terminator Cycle Sequencing Kit (ver. 3.1, Applied Biosystems) and an AB PRISM 3730 (Applied Biosystems) automatic sequencer.

### Distance and Phylogenetic Analyses

Forward and reverse sequences of COI and 16S rDNA were edited, assembled and merged into consensus sequences using the software program SeqmanII 5.07 (Lasergene, DNASTAR, Madison, WI, USA). Sequences were aligned using the program, fftnsi, which is implemented in MAFFT 6.717 [50]. Alignment of COI nucleotide sequences was unproblematic since indels were absent. For 16S rDNA, areas of uncertain alignment were omitted by the software Gblocks 0.91b [51], with minimum number of sequences for a conserved position set to 50% of the total, minimum number of sequences for a flanking position set to 90% of the total, maximum number of contiguous non-conserved positions set to 3, minimum length of a block set to 5, and half gap positions allowed.

For distance analyses, pairwise sequence divergences were calculated using a Kimura 2-parameter (K2P) distance model and analyzed at species, genus and family level in MEGA 4.0 [52] for COI and 16S rDNA respectively. Neighbour-joining (NJ) analyses were also conducted independently for COI and 16S rDNA datasets using K2P distance model as recommended by Hebert et al. [1] using MEGA 4.0 [52]. Node support was evaluated with 1000 bootstrap pseudoreplicates. *Cypraea cervinetta* (Cypraeidae) was selected as the outgroup.

### Character-Based Barcode Analysis

For the character-based identification method, we used the characteristic attribute organization system (CAOS) [53], [54]. The CAOS algorithm identifies character-based diagnostics, here termed “characteristic attributes” (CAs), for every clade at each branching node within a guide tree that is first produced from a given dataset. CAs are diagnostic character states (genes, amino acids, base pairs or even morphological, ecological or behavioural attributes) which are found only in one clade but not in an alternate group that descends from the same node [21]. The system comprises two programs: P-Gnome and P-Elf [53]. In this study, the programs PAUP v4.0b10 [55] and Mesquite v2.6 [56] were used to produce the input NJ trees and nexus files for P-Gnome respectively in accordance with the CAOS manual. The input tree for P-Gnome requires that all species nodes be collapsed to single polytomies. Then several sets of data were executed in P-Gnome. The most variable sites that distinguish all the taxa were chosen and the character states at these nucleotide positions were listed. Finally, unique combinations of character states (character-based DNA barcodes) were identified.

## Results

In total, we analysed 108 COI and 102 16S rDNA sequences from 113 neogastropod individuals (Table S1). Therein, 63 COI and 58 16S sequences were obtained from this study (Genbank accession numbers: JN052927–JN053047). Other 45 COI and 44 16S rDNA sequences were obtained from our previous studies [49]. For 97 individuals included, both COI and 16S rDNA sequences were obtained.

### Distance-Based Barcode

**Figure 1:**
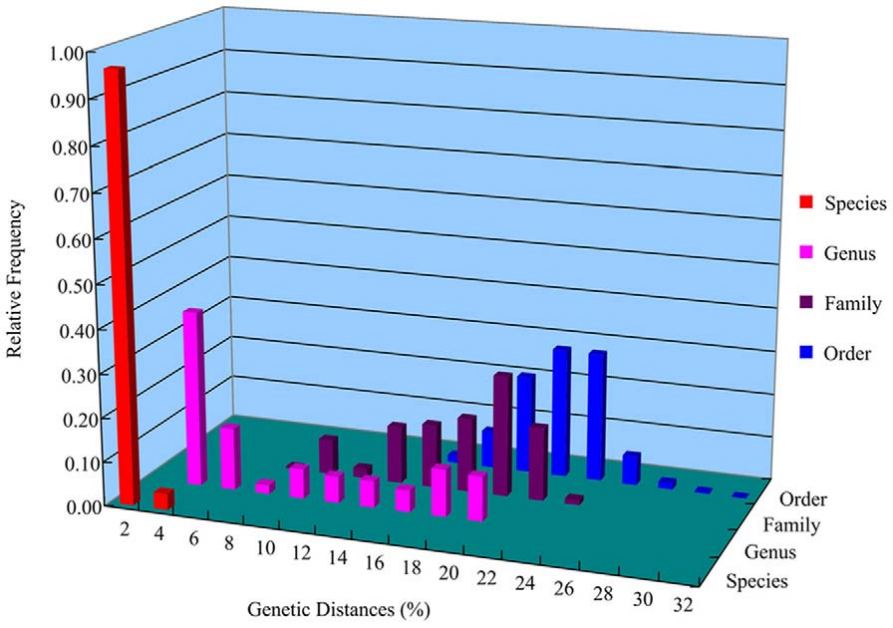
Distribution of genetic divergences based on the K2P distance model for COI sequences.

The genetic divergences of COI sequences according to different taxonomic levels within the order Neogastropoda were analyzed (Figure 1 and Table 1). As expected, genetic divergence increased with higher taxonomic rank. The pairwise genetic divergences among conspecific individuals ranged from 0% to 2.20% with a mean of 0.64%. Mean pairwise divergence between specimens of congeneric species was 8.06% (range. 2.10%–19.80%). Mean pairwise divergence between specimens of different genera that belong to the same family was 18.46% (range. 6.3%–24.80%), and mean pairwise divergence between specimens of different families that belong to Neogastropoda was 21.61% (range. 15.20%–30.90%). No “distance-gap” was found between intraspecific and interspecific divergences of COI sequences within Neogastropoda (Figure 1). The “10× rule” threshold (6.4% in this study) resulted in lumping 45% of distinct species. The less interspecific divergences (range. 2.10%–3.90%) were found between specimens of *Hemifusus* species (*Hemifusus colosseus*, *Hemifusus ternatanus* and *Hemifusus tuba*), and overlapped the intraspecific divergences.

**Table 1:** COI genetic divergences according to different taxonomic levels within the order Neogastropoda.

Comparison	Average (%)	Minimum (%)	Maximum (%)	SE
Within species	0.64	0.00	2.20	0.002
Within genus, between species	8.06	2.10	19.80	0.010
Within family, between genera	18.46	6.30	24.80	0.017
Within order, between families	21.61	15.20	30.90	0.022

**Figure 2:**
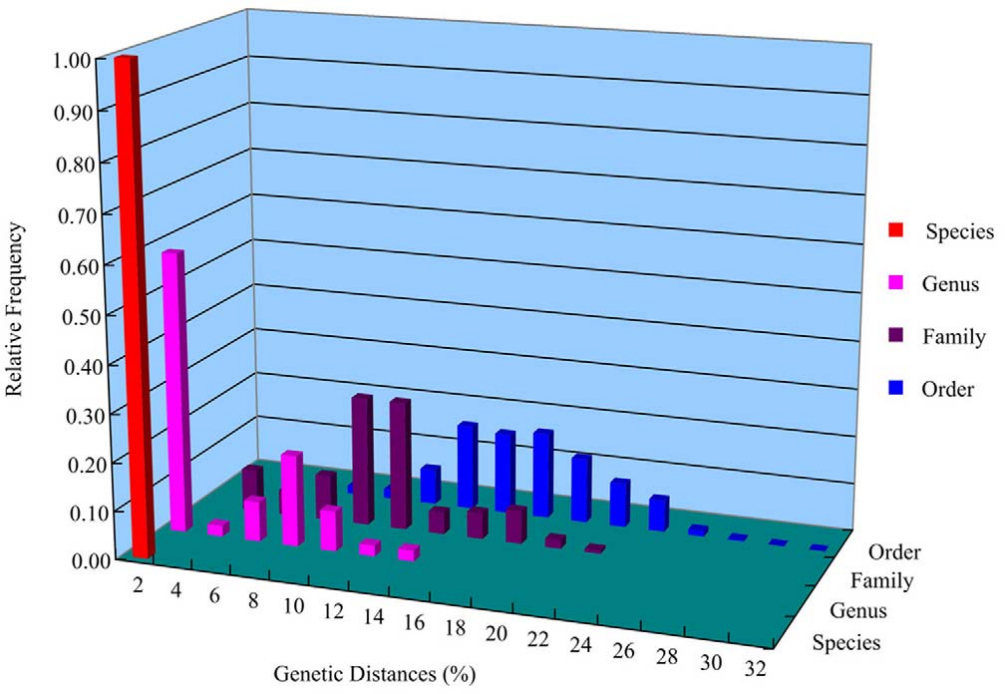
Distribution of genetic divergences based on the K2P distance model for 16S rDNA sequences.

The genetic divergences of 16S rDNA for different taxonomic levels within the order Neogastropoda were shown in Figure 2 and Table 2. The pairwise genetic divergences among conspecific individuals ranged from 0% to 1.60% with a mean of 0.20%. Mean pairwise divergence between specimens of congeneric species was 3.41% (range. 0.30%–12.40%). Mean pairwise divergence between specimens of different genera that belong to same family was 9.65% (range. 2.20%–21.70%), and mean pairwise divergence between specimens of different families that belong to Neogastropoda was 16.32% (range. 6.90%–30.20%). Thus, obvious overlap between intraspecific and interspecific divergences of 16S rDNA sequences was found (Figure 2). The “10× rule” threshold (2.0% in this study) resulted in lumping 57% of distinct species. Generally, the divergence at each taxonomic level for 16S rDNA gene was less than that for COI gene.

**Table 2:** 16S rDNA genetic divergences according to different taxonomic levels within the order Neogastropoda.

Comparison	Average (%)	Minimum (%)	Maximum (%)	SE
Within species	0.20	0.00	1.60	0.001
Within genus, between species	3.41	0.30	12.40	0.009
Within family, between genera	9.65	2.20	21.70	0.018
Within order, between families	16.32	6.90	30.20	0.024

### Neighbour-Joining Clusters

**Figure 3:**
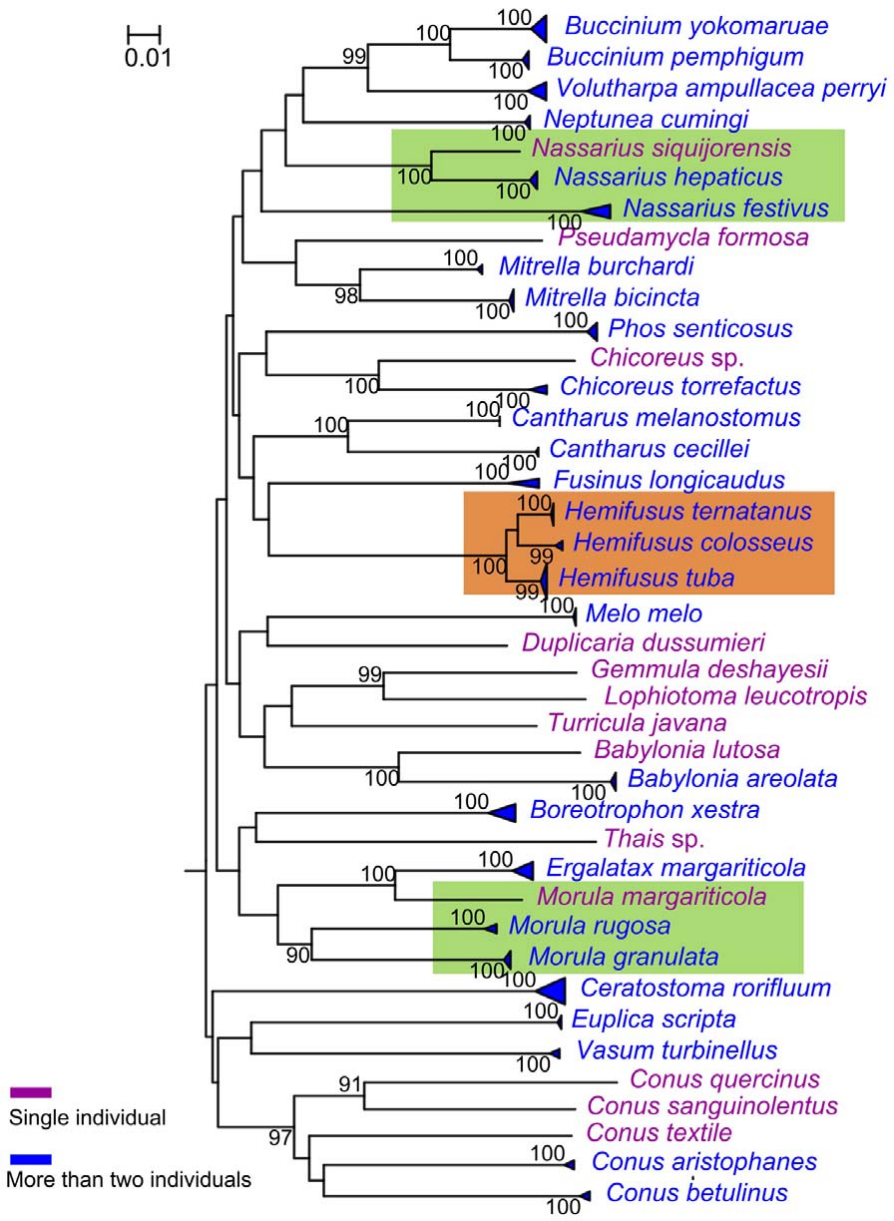
Neighbour-joining tree based on 108 COI sequences belonging to Neogastropoda from a wide phylogenetic range.

**Figure 4:**
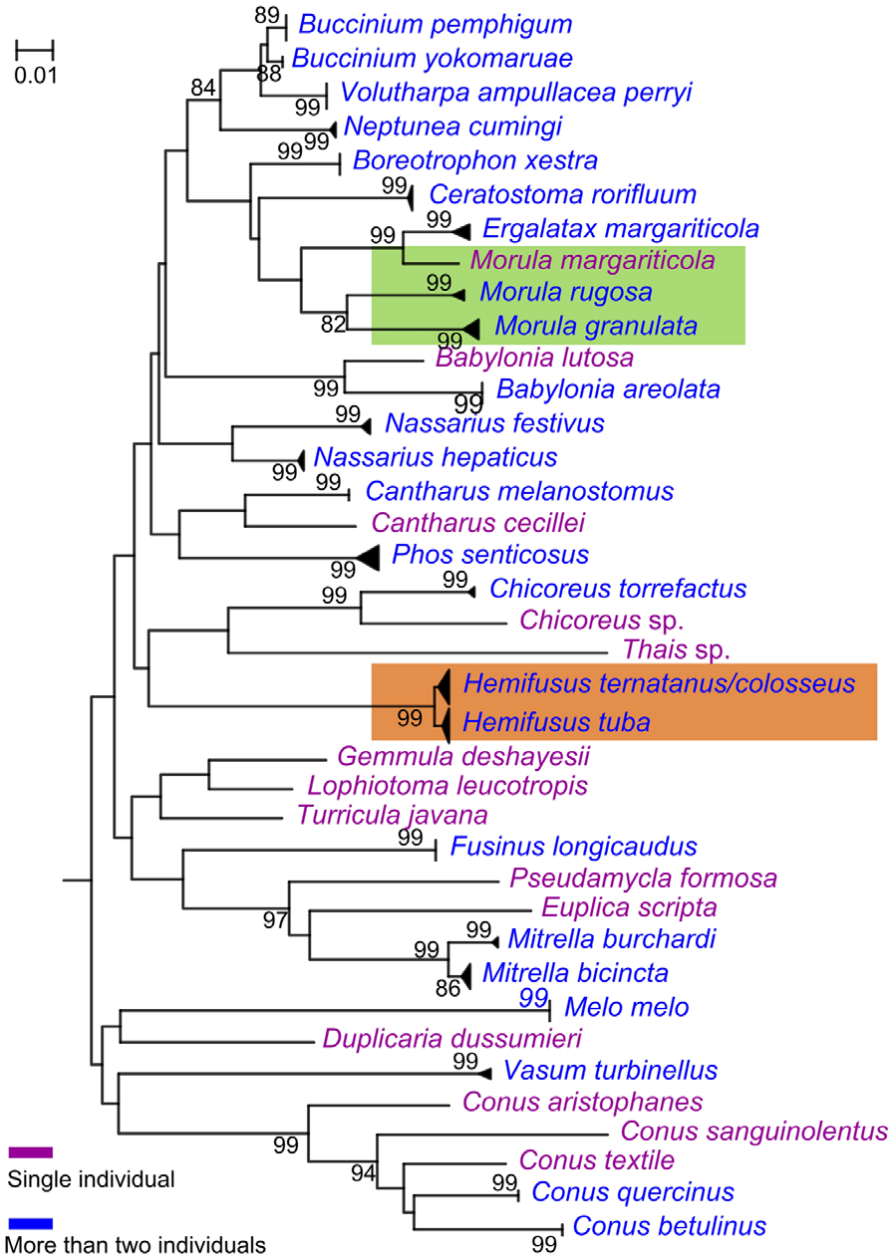
Neighbour-joining tree based on 102 16S rDNA sequences belonging to Neogastropoda from a wide phylogenetic range.

The COI NJ tree depicted all species where more than one individual were sequenced as monophyletic with 99% or 100% bootstrap support (Figure 3). No bootstrap support could be calculated for the species represented by a single individual. Three *Hemifusus* species showed close relationship in the COI tree (Figure 3). The species where more than one individual were sequenced were also shown as monophyletic clades in the 16S rDNA tree except the species *H. colosseus* and *H. ternatanus* that grouped into one cluster (Figure 4). Some species were less supported in the 16S rDNA tree (e.g., *Mitrella bicincta*). The genera *Nassarius* and *Morula* were not monophyletic in COI and 16S rDNA trees (Figures 3 and 4).

### Character-Based Barcode

#### (a) Species Level

In the COI gene region of the order Neogastropoda for 40 species character states at 29 nucleotide positions were found (Table S2). The particular nucleotide positions were chosen due to the high number of CAs at the important nodes or because of the presence of CAs for groups with highly similar sequences. All of the 40 species revealed a unique combination of character states at 29 nucleotide positions with at least three CAs for each species. For the closely related species *H. colosseus*, *H. ternatanus* and *H. tuba*, only 4 diagnostic characters were identified.

The character states at 27 nucleotide positions of the 16S rDNA gene region for 39 species of the order Neogastropoda were shown (Table S3). As the COI gene region resolved, all species revealed a unique combination of character states at 27 nucleotide positions with at least three CAs for each species. Only 3 diagnostic characters were detected among the species *H. colosseus*, *H. ternatanus* and *H. tuba*.

As less diagnostic characters were detected for the *Hemifusus* species (*H. colosseus*, *H. ternatanus* and *H. tuba*), we extracted character-based DNA barcodes from a subset of the three *Hemifusus* species. The character states at 30 nucleotide positions of the COI gene region were found with at least fourteen CAs for each species (Table S4). The character states at 21 nucleotide positions of the 16S rDNA gene region were also found with at least five CAs for each species (Table S5). The three *Hemifusus* species were clearly distinguished by the diagnostic characters of COI and 16S rDNA gene regions in the subset.

#### (b) Genus Level

The character states for 25 neogastropod genera at 32 nucleotide positions of the COI gene region were found (Table S6). Dashed cells indicated non-significant positions at which at least three different nucleotides occurred within a genus. Unique combinations of at least three diagnostic character states were found for 23 out of 25 genera. However, only one diagnostic character at position 419 (T→A) was found for the genera *Conus* and *Morula*.

The character states for 25 neogastropod genera at 32 nucleotide positions of the 16S rDNA gene region were also shown (Table S7). All the genera revealed a unique combination of character states at the 32 nucleotide positions with at least three CAs for each genus.

## Discussion

### Distance and Phylogenetic Assignments

The use of a distance-based threshold technique has been a major point of contention in the DNA barcoding [13], [19], [57]. While gene variation represents a product of evolution, an arbitrary cut-off value does not reflect what is known about the evolutionary processes responsible for this variation. In addition, the shortcoming of distance-based approach involves the lack of an objective set of criteria to delineate taxa. For example, a universal similarity cut-off to determine species status will simply not exist, because of the broad overlap of inter-and intraspecific distances [10]. In this study, the “barcoding gap” between levels of intraspecific variation and interspecific divergence does not exist in either analysis of COI or 16S rDNA sequences. On the contrary, obvious overlap between intraspecific and interspecific divergences is found in both COI and 16S rDNA analysis, especially in the 16S rDNA analysis. We find the original “10× rule” threshold proposed by Hebert et al. [33] to be too liberal to recognize the neogastropod species studied. The “10× rule” threshold can result in lumping about half of distinct species for both COI and 16S rDNA sequences in this study. The species which can not be discriminated by “10× rule” threshold are mostly closely related. Thus, we have to be cautious to use the distance-based threshold method for species identification, at least for Neogastropoda that include a great deal of recently divergent species. Finally, our analysis shows a general increase in the molecular divergence of COI and 16S rDNA sequences with taxonomic rank, a trend that suggests that morphological taxonomy is roughly in agreement with DNA evolution. Yet, this relationship is not entirely consistent, and the distribution of divergences at different taxonomic scales often overlaps.

The development of an NJ profile for identification (monophyly-based approach) depends on the coalescence of species and not an arbitrary level of divergence [20]; in theory, species that failed recognition via the threshold approach may still be recognized [58]. In this case study, the COI and 16S rDNA sequences produce different topologies. The COI NJ tree depicts all species where more than one individual are sequenced as monophyletic with strong support. However, in 16S rDNA tree, the closely related species *H. colosseus* and *H. ternatanus* group into one cluster and the monophyly of some species is weakly supported. The reason may be that 16S rDNA sequences are relatively more conserved than COI sequences [59]. The recently divergent taxa are not reciprocally monophyletic due to lack of time needed to coalesce. Additionally, some genera are not recovered as monophyletic in COI and 16S rDNA NJ trees.

Despite the superiority of monophyly-based method for species discrimination compared with distance-based method, critics have argued that the bootstrap test for monophyly is simply too conservative and incorrectly rejects monophyly in too many cases [60]. In addition, since identification does not hinge on monophyly [61] and the use of reciprocal monophyly as a criterion for species recognition is arbitrary [62], it seems best to avoid using monophyly-based method. Indeed, several studies have already shown the limitations of distance and monophyly-based methods to identify species (e.g., [23], [63], [64], [65], [66], [67]). Nevertheless, due to the computational strengths shared by distance and monophyly-based methods, both methods can still be used to flag species and compare with resolution of other identification methods. Especially, the monophyly-based method can be helpful for initial species identification.

### Character-Based DNA Barcoding

The character-based method of DNA barcoding is effective for the identification of genetic entities at species and genus levels in this study. On species level, both COI and 16S rDNA sequences can identify diagnostic barcodes for all species included. All species revealed a unique combination of character states at 29 and 27 nucleotide positions of COI and 16S rDNA gene regions respectively with at least three CAs for each species. Especially, the closely related species *H. colosseus* and *H. ternatanus* that can not be clearly distinguished by distance and monophyly-based methods show different diagnostic barcodes in both genes although with less CAs. Due to the less CAs, we extracted character-based DNA barcodes from a subset of three *Hemifusus* species. The results indicate that *H. colosseus*, *H. ternatanus* and *H. tuba* are clearly distinguished in both genes with more CAs for each species in the small subset. Since many closely related species are included in this study, we test the advantage of character-based method for distinguishing closely related species.

On the genus level, we find character-based barcodes with at least three CAs for 24 out of 25 genera in COI gene region and all genera in 16S rDNA gene region. However, only one diagnostic character is found for the genera *Conus* and *Morula* in COI sequences. The reason may be that there is a much higher level of interspecific variability in *Conus* and *Morula* COI sequences resulting in occurrence of more bases at each nucleotide position within a genus. While distance and monophyly-based approaches focus on the identification at species level, our study shows the suitability of character-based method for identification at genus level.

The establishment of reliable character-based DNA barcodes depends on the use of an appropriate genetic marker. The COI region of the mitochondrial genome has been the reference marker of choice in DNA barcoding studies [11], [68], [69], [70], [71], [72]. However, as more data have become available, a number of studies have experienced problems with the single locus approach (e.g. [73]). We show here that both COI and 16S rDNA genes are well suited as character-based barcode markers for neogastropod discrimination in species and genus level. Especially, the 16S rDNA sequences evolving slower than COI sequences show a better resolution of neogastropod identification by character-based method than by distance and monophyly-based methods. Thus, the character-based DNA barcoding can employ more sequence resource for species identification, even the relatively conserved genes. Goldstein and DeSalle [62] suggest that the legacy of DNA barcoding has the potential to extend far beyond a database of short sequences, towards a bank of genomic DNA. Character-based approach may provide chance for the use of genomic DNA for barcoding.

Another advantage of character-based barcoding is the fact that it is compatible with classical approaches allowing the combination of classical morphological and behavioral information. Contrary to phenetic barcoding (e.g., monophyly-based approach), the use of diagnostic characters has at its core the benefit of being visually meaningful, and better approximates a real barcode [74]. This is especially important to “integrative taxonomy” [62], [75] as species identification and discovery can be based on the combinations of molecular and traditional taxonomic information.

## Supporting Information

Figure S1 Sampling sites in this analysis.
(TIF)

Table S1 List of species DNA Barcoded in this study.
(DOC)

Table S2 Character-based DNA barcodes for COI gene for 40 neogastropod species at the species level.
(DOC)

Table S3 Character-based DNA barcodes for 16S rDNA gene for 39 neogastropod species at the species level.
(DOC)

Table S4 Character-based DNA barcodes for COI gene for 3 species belonging to the genus *Hemifusus*.
(DOC)

Table S5 Character-based DNA barcodes for 16S rDNA gene for 3 species belonging to the genus *Hemifusus*.
(DOC)

Table S6 Character-based DNA barcodes for COI gene at the genus level.
(DOC)

Table S7 Character-based DNA barcodes for 16S rDNA gene at the genus level.
(DOC)
