# Supplementary figures and images for: Comparing the Usefulness of Distance, Monophyly and Character-Based DNA Barcoding Methods in Species Identification: A Case Study of Neogastropoda
Source: PLoS One. 2011 Oct 24;6(10):e26619. doi: 10.1371/journal.pone.0026619 (PMC3200347; doi:10.1371/journal.pone.0026619)

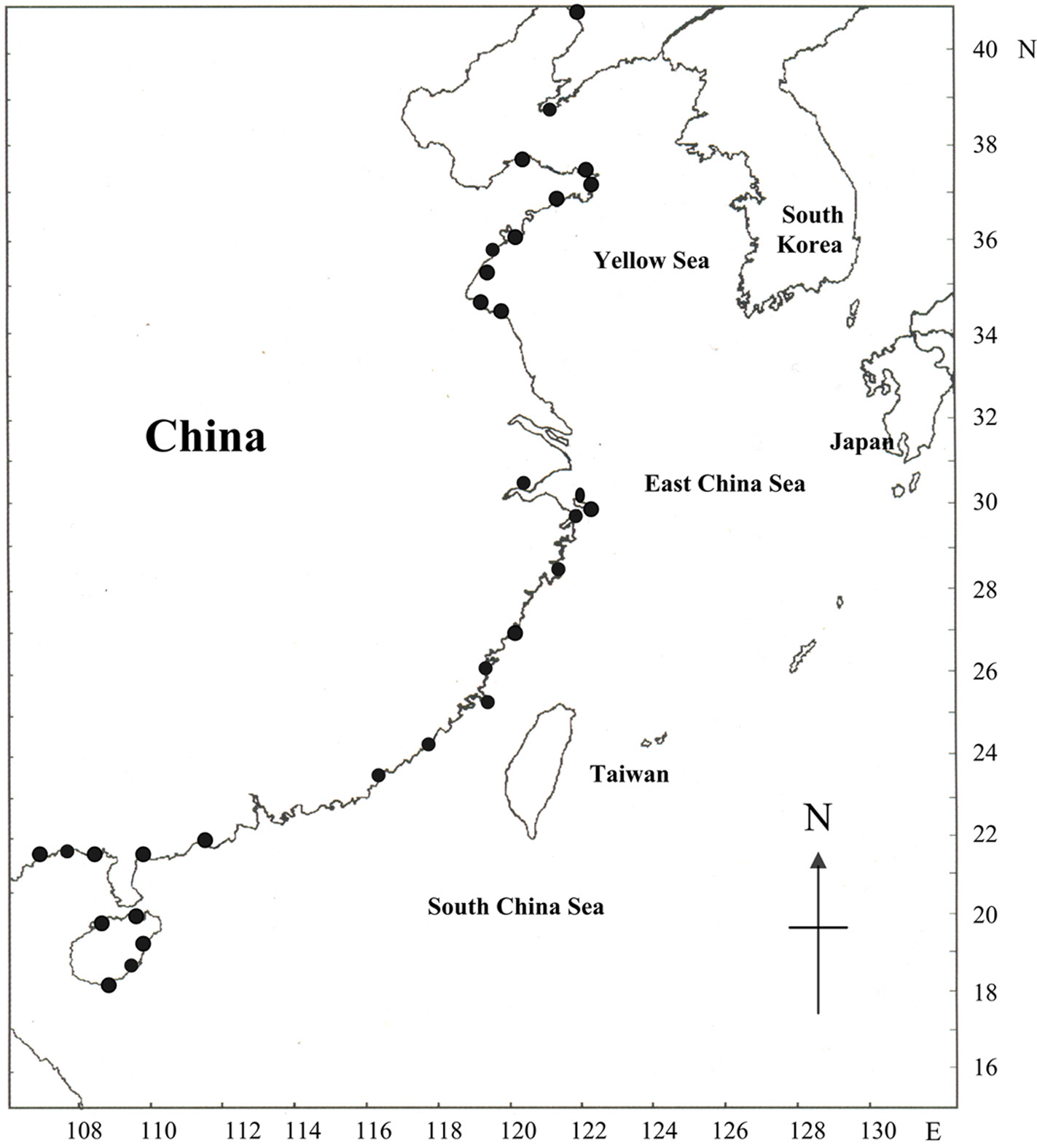

Supplement: Figure S1 — Sampling sites in this analysis. (TIF) [file pone.0026619.s001.tif]
