# Supplementary material for: Comparing the Usefulness of Distance, Monophyly and Character-Based DNA Barcoding Methods in Species Identification: A Case Study of Neogastropoda
Source: PLoS One. 2011 Oct 24;6(10):e26619. doi: 10.1371/journal.pone.0026619 (PMC3200347; doi:10.1371/journal.pone.0026619)
Supplement: Table S1 — List of species DNA Barcoded in this study. (DOC) [file pone.0026619.s002.doc]

Table S1. List of species DNA Barcoded in this study

| Family | Genus | Species | Abbreviation of species name | No. of  individuals |
| --- | --- | --- | --- | --- |
| Buccinidae | *Buccinium* | *pemphigum* | Bp | 6 |
|  |  | *yokomaruae* | By | 4 |
|  | *Phos* | *senticosus* | Ps | 5 |
|  | *Cantharus* | *melanostomus* | Cm | 2 |
|  |  | *cecillei* | Cc | 2 |
|  | *Volutharpa* | *ampullacea perryi* | Va | 5 |
|  | *Neptunea* | *cumingi* | Nc | 3 |
| Columbellidae | *Euplica* | *scripta* | Es | 3 |
|  | *Pseudamycla* | *formosa* | Pf | 1 |
|  | *Mitrella* | *bicincta* | Mb | 5 |
|  |  | *burchardi* | Mbu | 2 |
| Melongenidae | *Hemifusus* | *colosseus* | Hc | 2 |
|  |  | *ternatanus* | Ht | 5 |
|  |  | *tuba* | Htu | 8 |
| Muricidae | *Chicoreus* | sp. | Cs | 1 |
|  |  | *torrefactus* | Ct | 2 |
|  | *Boreotrophon* | *xestra* | Bx | 4 |
|  | *Thais* | sp. | Ts | 1 |
|  | *Ergalatax* | *margariticola* | Em | 2 |
|  |  |  |  | 2 |
|  | *Ceratostoma* | *rorifluum* | Cr | 2 |
|  |  |  |  | 4 |
|  | *Morula* | *rugosa* | Mr | 2 |
|  |  | *granulata* | Mg | 4 |
|  |  | *margariticola* | Mm | 1 |
| Nassariidae | *Nassarius* | *siquijorensis* | Zs | 1 |
|  |  | *hepaticus* | Nh | 4 |
|  |  | *festivus* | Nf | 3 |
| Fasciolariidae | *Fusinus* | *longicaudus* | Fl | 4 |
| Volutidae | *Melo* | *melo* | Mme | 4 |
| Babyloniidae | *Babylonia* | *lutosa* | Bl | 1 |
|  |  | *areolata* | Ba | 4 |
| Conidae | *Conus* | *aristophanes* | Ca | 2 |
|  |  | *textile* | Cte | 1 |
|  |  | *betulinus* | Cb | 2 |
|  |  | *quercinus* | Cq | 2 |
|  |  | *sanguinolentus* | Csa | 1 |
| Turbinellidae | *Vasum* | *turbinellus* | Vt | 2 |
| Terebridae | *Duplicaria* | *dussumieri* | Dd | 1 |
| Turridae | *Turricula* | *javana* | Tj | 1 |
|  | *Gemmula* | *deshayesii* | Gd | 1 |
|  | *Lophiotoma* | *leucotropis* | Ll | 1 |
