# Supplementary material for: Comparing the Usefulness of Distance, Monophyly and Character-Based DNA Barcoding Methods in Species Identification: A Case Study of Neogastropoda
Source: PLoS One. 2011 Oct 24;6(10):e26619. doi: 10.1371/journal.pone.0026619 (PMC3200347; doi:10.1371/journal.pone.0026619)

**Table S4**. Character-based DNA barcodes for 3 species belonging to the genus *Hemifusus*; Character states (nucleotides) at 30 selected positions of the COI gene region (ranging from position 14–641); taxa = abbreviations according to table S1; numbers of individuals analysed per species are given in brackets; When two character states were present at a given position the number of individuals showing each character is given in brackets.


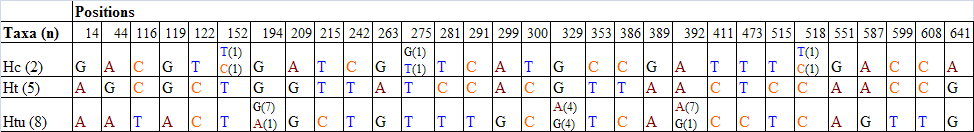

Supplement: Table S4 — Character-based DNA barcodes for COI gene for 3 species belonging to the genus Hemifusus. (DOC) [file pone.0026619.s005.doc]
