# Supplementary material for: Comparing the Usefulness of Distance, Monophyly and Character-Based DNA Barcoding Methods in Species Identification: A Case Study of Neogastropoda
Source: PLoS One. 2011 Oct 24;6(10):e26619. doi: 10.1371/journal.pone.0026619 (PMC3200347; doi:10.1371/journal.pone.0026619)

Table S7 Character-based DNA barcodes at the genus level: Character states (nucleotides) at 32 selected positions of the 16S rDNA gene region (ranging from 69 – 319); dashed cells indicate the occurrence of three or all four bases at this particular nucleotide position within a genus; cells were shaded grey if two different bases were present within a genus; numbers of analysed species and individuals are shown in brackets.


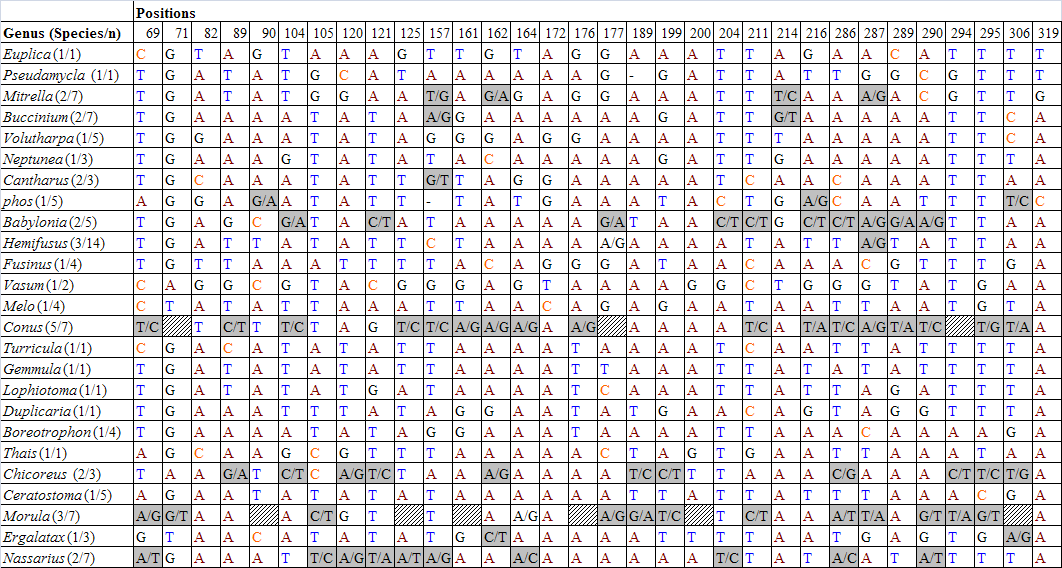

Supplement: Table S7 — Character-based DNA barcodes for 16S rDNA gene at the genus level. (DOC) [file pone.0026619.s008.doc]
